# Prognostic value of temporal patterns of left atrial reservoir strain in patients with heart failure with reduced ejection fraction

**DOI:** 10.1007/s00392-023-02244-x

**Published:** 2023-06-13

**Authors:** S. Abou Kamar, Y. S. Aga, M. de Bakker, V. J. van den Berg, M. Strachinaru, D. Bowen, R. Frowijn, K. M. Akkerhuis, J. J. Brugts, O. Manintveld, V. Umans, M. Geleijnse, R. A. de Boer, E. Boersma, I. Kardys, B. M. van Dalen

**Affiliations:** 1 Department of Cardiology, Thoraxcenter, Erasmus MC, University Medical Center Rotterdam, Room Na‐316, P.O. Box 2040, 3000 CA Rotterdam, The Netherlands; 2 The Netherlands Heart Institute, Utrecht, The Netherlands; 3 Department of Cardiology, Franciscus Gasthuis & Vlietland, Rotterdam, The Netherlands; 4 Department of Cardiology, Northwest Clinics, Alkmaar, The Netherlands; 5 Department of Anesthesiology, Leiden University Medical Center, Leiden, The Netherlands

**Keywords:** Left atrial reservoir strain, Heart failure with reduced ejection fraction, Longitudinal studies, Repeated measurements

## Abstract

**Background:**

We investigated whether repeatedly measured left atrial reservoir strain (LASr) in heart failure with reduced ejection fraction (HFrEF) patients provides incremental prognostic value over a single baseline LASr value, and whether temporal patterns of LASr provide incremental prognostic value over temporal patterns of other echocardiographic markers and NT-proBNP.

**Methods:**

In this prospective observational study, 153 patients underwent 6-monthly echocardiography, during a median follow-up of 2.5 years. Speckle tracking echocardiography was used to measure LASr. Hazard ratios (HRs) were calculated for LASr from Cox models (baseline) and joint models (repeated measurements). The primary endpoint (PEP) comprised HF hospitalization, left ventricular assist device, heart transplantation, and cardiovascular death.

**Results:**

Mean age was 58 ± 11 years, 76% were men, 82% were in NYHA class I/II, mean LASr was 20.9% ± 11.3%, and mean LVEF was 29% ± 10%. PEP was reached by 50 patients. Baseline and repeated measurements of LASr (HR per SD change (95% CI) 0.20 (0.10–0.41) and (0.13 (0.10–0.29), respectively) were both significantly associated with the PEP, independent of both baseline and repeated measurements of other echo-parameters and NT-proBNP. Although LASr was persistently lower over time in patients with PEP, temporal trajectories did not diverge in patients with versus without the PEP as the PEP approached.

**Conclusion:**

LASr was associated with adverse events in HFrEF patients, independent of baseline and repeated other echo-parameters and NT-proBNP. Temporal trajectories of LASr showed decreased but stable values in patients with the PEP, and do not provide incremental prognostic value for clinical practice compared to single measurements of LASr.

**Graphical abstract:**

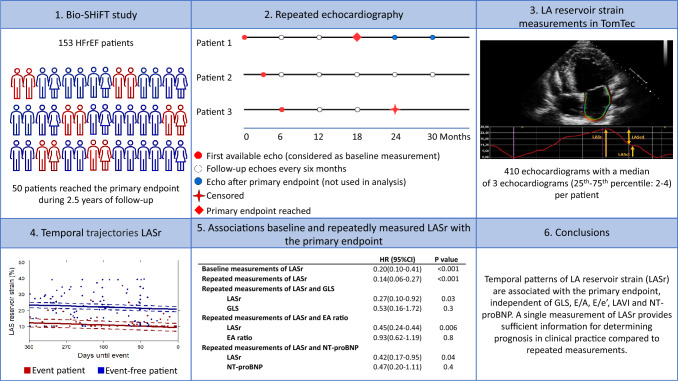

**Supplementary Information:**

The online version contains supplementary material available at 10.1007/s00392-023-02244-x.

## Introduction

Most of the contemporary risk scores for heart failure with reduced ejection fraction (HFrEF) focus on systolic echocardiographic determinants, while the influence of diastolic determinants on prognosis has been studied less extensively [1]. Categorization of HFrEF patients based on diastolic determinants is mainly used to non-invasively estimate left atrial pressure (LAP), which can be useful to guide medical treatment and provide information on prognosis [2, 3]. However, the algorithm currently in use for estimating LAP carries an important limitation; it requires multiple parameters that are often affected by cardiac rhythm and/or mitral valve disease, with the consequence that a substantial part of HFrEF patients remain uncategorized [4].

Recently, there has been an emerging interest in the use of left atrial reservoir strain (LASr) as a measure of left atrial (LA) function and as a derived measure for LAP in HFrEF patients [5–8]. LASr is predominantly determined by LV GLS and filling pressure [7]. Although LA strain could have limitations like load dependency, Doppler echocardiography may carry more limitations. It is angle-dependent, which is not an issue in strain analysis [9]. In addition, LA strain is preload dependent, but to a lesser degree than LA volume [10]. Studies have demonstrated that LASr is decreased in HFrEF patients and that an abnormal LASr is associated with increased LAP [7, 8]. Additionally, studies have shown that LASr carries prognostic information in patients with atrial fibrillation and mitral valve disease. This implies that LASr is less affected by these conditions as compared to conventional echocardiographic parameters, to estimate LAP [11, 12]. Only a few studies have demonstrated that LASr may have prognostic value in HFrEF [13–15]. These studies only examined single measurements of LASr, which merely represent a snapshot of a patient’s condition, and related these measurements to clinical endpoints occurring over several years thereafter. The prognostic value of repeatedly measured LASr has never been examined before and has never been compared to that of other echocardiographic parameters in chronic HFrEF patients. 

Therefore, we investigated whether repeatedly measured LASr provides incremental prognostic value over a single baseline LASr value in stable chronic HFrEF patients. In addition, we hypothesized that temporal patterns of LASr are associated with adverse clinical events, and that temporal patterns of LASr may provide incremental prognostic value to temporal patterns of other prognostic markers.

## Methods

### Study design

The design of the Serial Biomarker Measurements and New Echocardiographic Techniques in Chronic Heart Failure Patients Result in Tailored Prediction of Prognosis (Bio-SHiFT) study has previously been described [16]. Bio-SHiFT is a prospective, observational study of stable patients with chronic heart failure (CHF), conducted at the Erasmus MC, Rotterdam, and Northwest clinics, Alkmaar, The Netherlands. Recruitment was conducted during the patient's regular outpatient visits while in clinically stable condition (i.e., they had not been hospitalized for HF in the 3 months prior to inclusion). The main inclusion criteria were diagnosis of HF according to the then prevailing guidelines of the European Society of Cardiology 3 or more months before inclusion and age ≥ 18 years [17]. Patients with an atrial pacemaker were excluded from the current analysis. Patients were observed for a maximum of 30 months, with follow-up visits scheduled every 3 months. A brief medical examination and blood samples were taken at each visit. All patients’ usual outpatient follow-up with their treating physician continued throughout the study, independently of the study visits. This study was approved by the medical ethics committees, conducted in accordance with the Declaration of Helsinki, and registered in ClinicalTrials.gov (NCT01851538). Informed consent was obtained from all patients. In total, 398 patients were included in Bio-SHiFT. The repeated echo study that we currently report was performed at the Erasmus MC only and consisted of 175 HFrEF patients with echocardiographic assessment every 6 months during follow-up [18]. Two patients had insufficient image quality, and therefore the remaining 173 patients were included in the current study.

### Echocardiography measurements and evaluation

Two-dimensional gray-scale harmonic images were obtained in the left lateral decubitus position. Standard apical four-, three-, and two-chamber views were recorded. A commercially available ultrasound system was used (iE33, Philips, Best, The Netherlands), equipped with a broadband (1–5 MHz) S5-1 transducer (frequency transmitted 1.7 MHz, received 3.4 MHz). Images were stored in the echo core lab of Erasmus MC. Using specialized software (2D Cardiac Performance Analysis version 4.5; TomTec Imaging Systems, Unterschleissheim, Germany), LVEF, tricuspid regurgitation (TR) velocity, and the function of the mitral valves were assessed according to the then prevailing guidelines [4]. The diastolic parameters were evaluated using Philips Excellera version R4.1 (Philips Medical Systems, The Netherlands) or TomTec Imaging Systems. To assess diastolic function indexed left atrial volume (LAVI), the peak early filling velocity (*E*)/late filling velocity (*A*) ratio (*E*/*A* ratio) and the ratio of the E and early diastolic mitral annular velocity (*e*’) (*E*/*é* ratio) were calculated [4]. For the *e*’, we used the mean of the lateral and medial *e*’ when available; however, if only one of the two was available, this value was used. All echocardiographic measurements were performed blinded to biomarker and clinical event data [16].

**Fig. 1:**
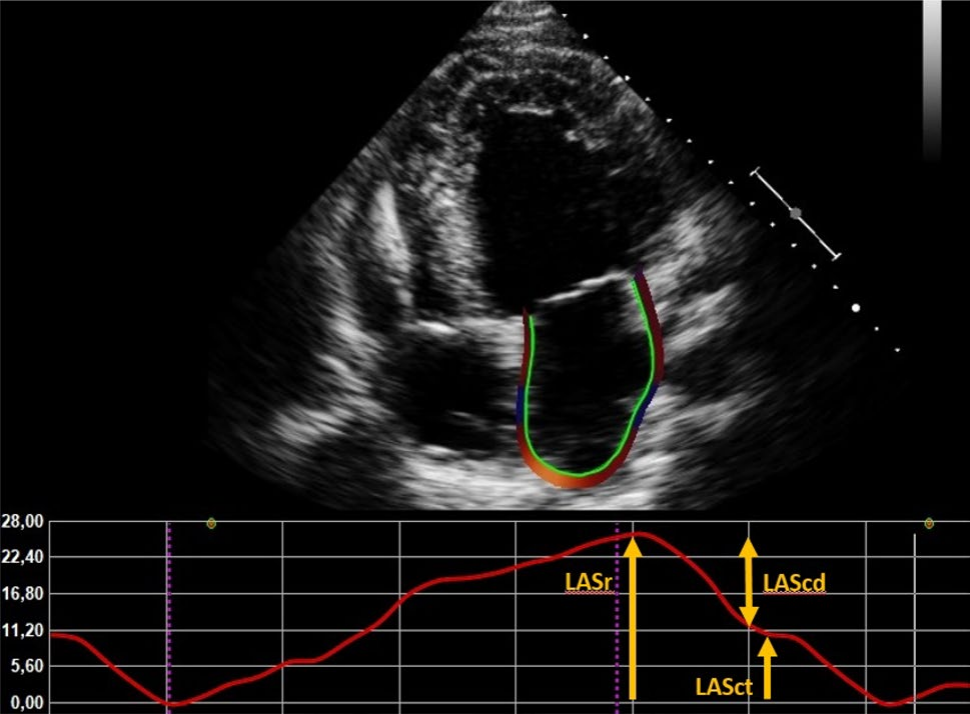
Example of LA strain measurement. *LASr* left atrial reservoir strain, *LAScd* left atrial conduit strain, *LASct* left atrial contractile strain

Strain parameters were measured with speckle tracking echocardiography (also using TomTec Imaging Systems) by a single operator, according to the prevailing consensus document of the EACVI/ASE/Industry Task Force to standardize deformation imaging [17]. The apical 4-chamber view was used preferably for the analysis. LA endocardial borders were automatically traced. The LA is contoured extrapolating across the pulmonary veins and LA appendage orifice [19]. We used end-diastole as a reference. Fine-tuning was performed manually if the tracking was suboptimal. If the quality of the 4-chamber view was poor, the 2-chamber view was used. Patients with insufficient image quality to perform LA strain analysis or patients with an atrial pacemaker were excluded. LA strain was assessed according to the three phases of the LA cycle: LA reservoir strain (LASr) which starts at the end of ventricular diastole (mitral valve closure) and continues until mitral valve opening (20). An example of a LA strain curve is provided in Fig. 1. Global longitudinal strain (GLS) was assessed in 18 LV segments on the standard apical four-, three-, and two-chamber views, where the endocardial border was traced manually at end-systole. The mean GLS from the three apical views was considered the LV GLS. If a patient had AF during the echocardiography, the index beat method was used. This is a validated method to measure echocardiographic parameters during AF [21].

**Fig. 2:**
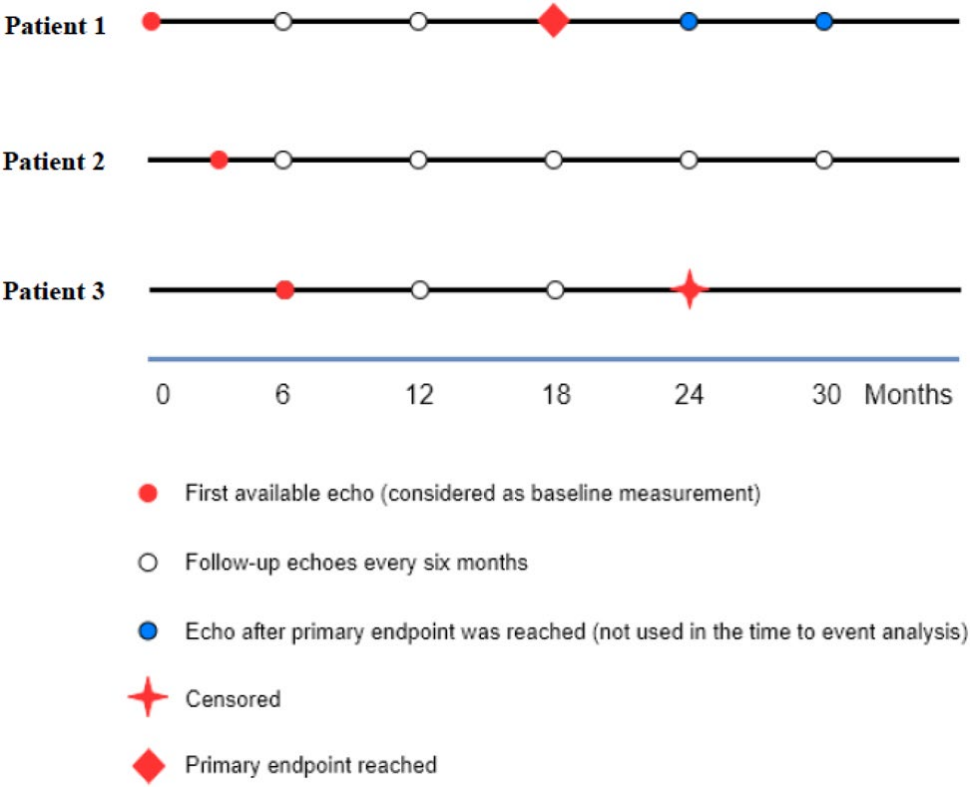
Study design: first available and follow-up echocardiograms. This figure provides 3 example patients to illustrate which echocardiograms were the first available echocardiograms, considered as ‘baseline’ in the analysis (red circles), and at which time-points follow-up echocardiograms were scheduled (white circles). 55% of the first available echocardiograms were performed at baseline (follow-up time zero), 12.8% were performed during the first study follow-up visit (target follow-up time 3 months) and 18% were performed during the second follow-up visit (target 6 months). Subsequently, echoes were performed every six months

Patients underwent echocardiographic assessment at baseline and every 6 months during follow-up. Due to logistic reasons, 58% of the first available echoes were performed at baseline (follow-up time zero), 15% of the first available echoes were performed within 3 months after the start of the study, 18% within 6 months after the start of the study, and the remaining 9% thereafter (Fig. 2). Missing echocardiograms occurred due to logistic circumstances (e.g., the unavailability of an ultrasound technician during the study visit). Intra-observer reproducibility was assessed by re-measuring GLS in 20 echocardiograms and calculating the intraclass correlation coefficient. The intraclass correlation coefficient was 0.93 for LASr.

### Clinical study endpoints

The primary endpoint (PEP) comprised the composite of hospitalization for the management of acute or worsened HF, left ventricular assist device (LVAD) implantation, cardiac transplantation, and cardiovascular death, whichever occurred first in time. All events were adjudicated by a clinical event committee blinded to the echocardiographic assessments and biomarker measurements, after reviewing corresponding hospital records and discharge letters [18].

### Statistical analyses

Distributions of continuous variables were tested for normality using the Shapiro–Wilk test. Normally distributed continuous variables are presented as mean ± standard deviation (SD), and non-normally distributed variables as a median and 25th–75th percentile. Categorical variables are presented as numbers and percentages. Differences in baseline characteristics between patients who experienced the PEP and those who did not were tested using the *t*-test and Mann–Whitney test, according to variable distributions, for continuous variables. For categorical variables, *χ*^2^-tests and Fisher’s exact tests were used.

First, we examined single measurements of LASr and other echo parameters of interest in relation to the PEP using Cox models (only the first available echo was used), and we adjusted for age, sex, duration of HF and N-terminal pro-b-type natriuretic peptide (NT-proBNP). In addition, we calculated the Pearson correlation coefficients for the echo variables of interest to assess the correlation with LASr.

Then, we assessed the value of repeated strain measurements for prediction of the PEP, as well as their incremental value to sole, baseline measurements. For this purpose, joint models for longitudinal and survival data were used [22]. In these joint models, a linear mixed effects (longitudinal) model provided estimates of the individual temporal trajectories of the echo parameters, and in combination with a relative risk model, the association of the trajectories with the risk of the PEP was estimated. The associations between the temporal evolutions of LASr and the PEP, resulting from the relative risk model, were first only adjusted for age, sex and duration of HF (model 1). Thereafter, baseline NT-proBNP (model 2), GLS and LVEF (model 3) were added consecutively. Furthermore, we adjusted for the diastolic parameters (E/A ratio, E/e ratio, LAVI) in model 4. Lastly, all variables with significant differences between those with and without the PEP were added (model 5). To investigate the incremental value of repeatedly measured LASr to repeatedly measured echo parameters and NT-proBNP, multivariable joint models were used.

We performed the above-described analyses on the full cohort. Subsequently, because LASr could be affected by the presence of AF and mitral regurgitation (MR), we performed subgroup analyses in patients with and without AF and MR.

To enable comparisons of effect sizes of different variables, we calculated the *Z*-scores for all investigated echo parameters, and NT-proBNP. Hazard ratios were obtained from both the Cox as the joint models. Thus, the results of the regression analyses of the Cox and joint models can be directly compared and are presented as HRs, which represent a risk per *Z*-score unit, along with the corresponding 95% confidence interval (CI).

Missing values in LASr and the other echo parameters (besides the A wave) were due to poor image quality and were therefore considered missing completely at random. Accordingly, we chose to perform a complete case analysis. Missing values for the A wave were due to atrial fibrillation during the echo or due to mitral valve replacement or clipping. In these patients the A wave can never be measured, thus imputation of missing values is inappropriate. Therefore, we again chose a complete case analysis here.

All analyses were performed with R Statistical Software using packages nlme [23] and JMbayes [22]. All tests were two-tailed, and *p* values < 0.05 were considered statistically significant.

## Results

### Baseline characteristics

Between October 2011 to January 2018, 173 patients were included in the Bio-SHiFT echo study. Twenty patients had an atrial pacemaker and were therefore excluded from the current analysis. Of the remaining 153 patients, 76% of the patients were male, mean age was 58 ± 11 years, and mean BMI was 27.5 ± 4.6 kg/m^2^. A total of 27% were in NYHA class I, and 55% were in NYHA class II. Ischemic heart disease was the most prevalent HF etiology (44%). The median time between diagnosis of HF and inclusion in the study was 6.5 (6.1–7.3) years. During a median follow-up time of 2.5 (2.3–2.6) years, a total of 50 patients (33%) reached the PEP, out of whom 37 were re-hospitalized for acute or worsened HF, 6 patients received a heart transplantation, 4 patients received an LVAD, and 3 patients died from a cardiovascular cause. Patients who reached the composite PEP had lower systolic and diastolic blood pressure, had a higher NT-proBNP (303 [180–540] vs. 71 [26–166] pmol/L, *p* < 0.001), and comorbidities such as atrial fibrillation and renal failure were more prevalent in this group (resp. 46% vs. 21%, *p* = 0.009; 58% vs. 30%, *p* = 0.003) (Table 1).

**Table 1:** Baseline patient characteristics of the total study population

	Overall	No PEP	PEP	*p* value
*N*	153	103	50	
Demographics				
Male, *n* (%)	116 (76)	79 (76)	37 (73)	0.9
Age, years	57.7 ± 11.2	57 ± 11.3	60(11.1)	0.2
Clinical characteristics				
Duration of HF, years	6.5 (6.1–7.3)	6.2 (5.9–6.9)	8.1 (7.0–9.3)	0.01
Body mass index, kg/m^2^	27.5 ± 4.7	27.8 ± 4.9	26.9 ± 4.2)	0.3
Mean heart rate, bpm	67 ± 13	67.2 ± 15.3	67.1 ± 8.0	1
Systolic blood pressure, mmHg	107 ± 18	110 ± 18	101 ± 17	0.008
Diastolic blood pressure, mmHg	67 ± 9	68 ± 9	64 ± 8	0.009
NYHA class, *n* (%)				0.06
NYHA class I	40 (27)	34 (33)	6 (10)	
NYHA class II	84 (55)	54 (53)	30 (60)	
NYHA class III	27 (18)	14 (14)	13 (26)	
NT-proBNP, pmol/L	141 (35–279)	71 (26–166)	303 (180–540)	< 0.001
Features of HF, *n* (%)				
Ischemic heart disease	67 (44)	42 (41)	25 (50)	0.3
Hypertension	2 (1)	2 (2)	0 (0)	0.8
Cardiomyopathy	58 (38)	38 (37)	20 (40)	0.9
Secondary to valvular heart disease	4 (3)	2 (2)	1 (2)	1
Other etiology of HF	13 (8)	11 (11)	2 (1)	1
Unknown	9 (6)	8 (8)	1 (2)	0.3
Medical history, *n* (%)				
Myocardial Infarction	65 (43)	40 (39)	24 (48)	0.3
PCI	58 (38)	39 (38)	18 (38)	1
CABG	15 (10)	10 (10)	5 (10)	1
Atrial fibrillation	46 (30)	22 (21)	22 (46)	0.009
Diabetes mellitus	37 (24)	23 (22)	13 (27)	0.8
Chronic renal failure	61 (40)	31 (30)	28 (58)	0.003
COPD	22 (14)	14 (14)	8 (1)	0.8
Medication use, *n* (%)				
Beta blockers	145 (95)	99 (96)	46 (92)	0.5
ACE inhibitors	106 (69)	75 (73)	33 (66)	0.4
Angiotensin II receptor blockers	43 (28)	27 (26)	14 (28)	0.9
Loop diuretics	143 (94)	93 (90)	50 (100)	0.04
Aldosterone antagonists	110 (71)	69 (67)	41 (82)	0.06

Normally distributed data are presented as mean ± SD, non-normally distributed data are presented as median (25th–75th percentile). *p* values represent an overall comparison between PEP and no PEP

*PEP* primary endpoint, *HF* heart failure, *NYHA* new york heart association, *PCI* percutaneous coronary intervention, *CABG* coronary artery bypass graft, *COPD* chronic obstructive pulmonary disease, *ACE* angiotensine converting enzyme

### Echocardiographic characteristics

During a median follow-time of 2.5 years, 410 echocardiograms were performed with a median of 3 (2–4) per patient. Patients had up to 8 consecutive echocardiographic evaluations performed, with 65% having at least 3 evaluations. An overview of the characteristics of the first available echocardiogram for each patient is presented in Table 2.

**Table 2:** Echocardiographic characteristics of the first available echo in relation to the composite endpoint

	Overall	No PEP	PEP	*p* value	Missing values
Left atrial strain					
LASr, %	20.9 ± 11.3	25.3 ± 10.5	11.7 ± 6.6	< 0.001	13 (3%)
LAScd, %	10.0 (7.2–15.5)	12.5 (8.8–17.4)	7.9 (3.75–9.22)	< 0.001	13 (3%)
LASct, %	9.3 (3.2–14.4)	12.0 (7.1–16.0)	2.8 (1.8–5.7)	< 0.001	13 (3%)
Systolic parameters					
LV GLS, %	− 9.0 ± 3.7	− 10.2 ± 3.6	− 6.4 ± 2.4	< 0.001	10 (2%)
LVEF, %	29.1 ± 10.4	31.6 ± 9.8	23.2 ± 9.3	< 0.001	10 (2%)
LV end-systolic diameter, mm	58.5 ± 11.7	55.9 ± 10.2	64.7 ± 12.8	0.001	15 (4%)
LV end diastolic diameter, mm	67.2 ± 10.5	64.9 ± 8.9	72.6 ± 12.3	0.002	15 (4%)
End-diastolic septal wall thickness, mm	8.6 ± 2.8	6.4 ± 1.9	9.7 ± 2.9	0.06	15 (4%)
End-diastolic posterior wall thickness, mm	9.0 ± 3.4	9.1 ± 1.2	9.0 ± 4.7	0.4	15 (4%)
Diastolic parameters					
LA volume index (min), mL/m^2^	25.4 ± 16.1	19.5 ± 11.8	37.9 ± 17.0	< 0.001	15 (4%)
LA volume index (max), mL/m^2^	39.1 ± 17.3	34.3 ± 14.3	49.3 ± 18.7	< 0.001	15 (4%)
LA emptying fraction, %	38.7 ± 17.9	45.6 ± 15.1	23.9 ± 14.6	< 0.001	15 (4%)
LA end-systolic volume, mL	78.4 ± 34.3	69.5 ± 29.7	97.4 ± 36.0	< 0.001	15 (4%)
LA end diastolic volume, mL	50.9 ± 31.4	39.8 ± 24.4	74.6 ± 31.9	< 0.001	15 (4%)
*E/A* ratio	1.4 ± 1.0	1.2 ± 0.9	2.1 ± 1.1	< 0.001	44 (11%)
*E/e*’ ratio	15.6 [9.5–19.7]	12.8 [7.9–19.2]	22.0 [12.9–24.0]	< 0.001	18 (4%)
TR velocity, m/s	2.5 (2.1–2.9)	2.4 (2.1–2.7)	2.7 (2.2–3.1)	0.1	50 (12%)
Mitral valve regurgitation, *n* (%)				0.001	11 (3%)
None	50 (33)	44 (45)	6 (14)		
Mild	60 (39)	35 (36)	25 (58)		
Moderate	23 (15)	16 (17)	7 (16)		
Severe	7 (5)	2 (2)	5 (12)		

Normally distributed data are presented as mean ± sd, non-normally distributed data are presented as median (25th–75th percentile). *p* values represent overall comparison between PEP and no PEP

*PEP* primary endpoint, *LASr* left atrial reservoir strain, *LAScd* left atrial conduit strain, *LASct* left atrial contractile strain, *LV* GLS left ventricular global longitudinal strain, LVEF left ventricular ejection fraction, *E/A* ratio the ratio of the peak early left ventricular filling velocity over the late filling velocity, *E/e*’ ratio *E* to early diastolic mitral annular tissue velocity, *TR* tricuspid regurgitation

Mean left ventricular ejection fraction (LVEF) in the total study population was 29.1% ± 10.4%, and mean LASr was 20.9% ± 11.3%. Patients who reached the PEP had significantly worse LASr compared to patients who remained PEP-free (LASr 11.7 ± 6.6% vs. 25.3 ± 10.5%, *p* < 0.001;. LVEF and GLS were also lower in patients who reached the PEP (resp. 23.2 ± 9.3% vs. 31.6 ± 9.8%, *p* < 0.001; − 6.4 ± 2.4% vs. − 10.2 ± 3.6%, *p* < 0.001). LAVI, E/A ratio and E/e’ were significantly higher in the PEP group (Table 2).

There was an inverse correlation between LASr and GLS (*r* = − 0.74, *p* < 0.001), E/A ratio (*r* = − 0.52, *p* < 0.001), *E*/*e*’ ratio (*r* = − 0.5, *p* < 0.001), LAVI (*r* = − 0.55, *p* < 0.001), and NT-proBNP (*r* = − 0.61, *p* < 0.001) (Supplementary Fig. 1).

### Baseline and repeatedly measured LASr in relation to the composite endpoint

When entered into separate models, baseline measurements of GLS, *E/e*’ ratio, and LASr were significantly associated with the PEP, independent of age, sex, duration of HF, and NT-proBNP, with the largest effect per one unit increase for LASr (resp. HR [95% CI] 0.46 [0.28–0.72]; 0.56 [0.37–0.84]; 0.20 [0.10–0.41]) (Table 3). Longitudinally measured LASr was significantly associated with the PEP in all the fitted joint models (Table 3). In the first model, adjusted for age, sex, and duration of HF, the HR(95% CI) was 0.19 (0.11–0.32). The association remained significant when NT-proBNP was added (HR [95% CI] 0.14 [0.06–0.27]). In model 3, GLS and LVEF were added as well (HR [95% CI] 0.21 [0.12–0.33]), and the association also remained significant in model 4, in which we adjusted for diastolic parameters (HR [95% CI] 0.13 [0.10–0.29]). The association between LASr and the PEP persisted in model 5 after adjusting for comorbidities (HR [95%CI] 0.19 [0.09–0.25]).

**Table 3:** Associations of baseline and repeatedly measured LASr with the primary endpoint

	HR (95%CI)	*p* value
Baseline measurements*		
LASr	0.20 (0.10–0.41)	< 0.001
GLS	0.46 (0.28–0.76)	0.003
LAVI	0.78 (0.58–1.05)	0.1
*E/A* ratio	0.66 (0.48–0.90)	0.01
*E/e*’ ratio	0.56 (0.37–0.84)	0.01
Repeated measurements of LASr		
Model 1	0.19 (0.11–0.32)	< 0.001
Model 2	0.14 (0.06–0.27)	< 0.001
Model 3	0.21 (0.12–0.33)	< 0.001
Model 4	0.13 (0.10–0.29)	< 0.001
Model 5	0.19 (0.09–0.25)	< 0.001
Repeated measurements of LASr and GLS, LAVI or *E/e*’ ratio†		
LASr and GLS		
LASr	0.27 (0.10–0.92)	0.038
GLS	0.53 (0.16–1.72)	0.3
LASr and LAVI		
LASr	0.47 (0.25–0.79)	0.004
LAVI	0.59 (0.46–1.45)	0.6
LASr and *E/A* ratio		
LASr	0.45 (0.24–0.44)	0.006
*E/A* ratio	0.93 (0.62–1.19)	0.8
LASr and *E/e*’ ratio		
LASr	0.56 (0.31–0.95)	0.03
*E/e*’ ratio	0.90 (0.62–1.43)	0.4
LASr and NT-proBNP		
LASr	0.42 (0.17–0.95)	0.04
NT-proBNP	0.47 (0.20–1.11)	0.4

*LASr* left atrial reservoir strain, *GLS* global longitudinal strain, *LAVI* left atrial volume indexed, *E/A* ratio the ratio of the peak early left ventricular filling velocity over the late filling velocity *E/e*’ ratio *E* to early diastolic mitral annular tissue velocity

*Corrected for age, sex, duration of HF, baseline NT-proBNP

^†^Multivariable Joint Models: Corrected for age, sex, duration of HF, atrial fibrillation, renal failure, systolic and diastolic blood pressure

Model 1: corrected for age, sex, duration of HF

Model 2: corrected for age, sex, duration of HF, NT-proBNP

Model 3: corrected for age, sex, duration of HF, NT-proBNP, GLS, LVEF

Model 4: corrected for age, sex, duration of HF, NT-proBNP, *E/A* ratio, *E/e* ratio, LAVI

Model 5: corrected for age, sex, duration of HF, atrial fibrillation, renal failure, systolic and diastolic blood pressure

The results of the multivariable joint models, wherein repeatedly measured LASr, as well as the other repeatedly measured echocardiographic variables were entered, are shown in Table 3. The HR for repeatedly measured LASr remained significant when correcting for repeatedly measured GLS, LAVI, *E/A* ratio, *E/e’* ratio, and NT-proBNP (resp. HR [95% CI] 0.27 [0.10–0.92]; 0.47 [0.25–0.79]; 0.45 [0.24–0.44]; 0.56 [0.31–0.95]; 0.42 [0.17–0.95]).

### Subgroup analyses in patients with atrial fibrillation and in patients with mitral regurgitation

The subgroup analyses showed that the repeated measurements of LASr were still significantly associated with the endpoint both in patients with SR as patients with AF, with an HR (95% CI) of 0.15 (0.06–0.34) and 0.03 (0.01–0.27), respectively (corrected for age, sex, duration of HF). This was also the case in patients with MR compared to patients with no MR, with HRs (95% CI) of 0.09 (0.03–0.22) and 0.14 (0.06–0.33), respectively. The full results are presented in Supplementary Table 1.

### Temporal evolution of LASr

**Fig. 3:**
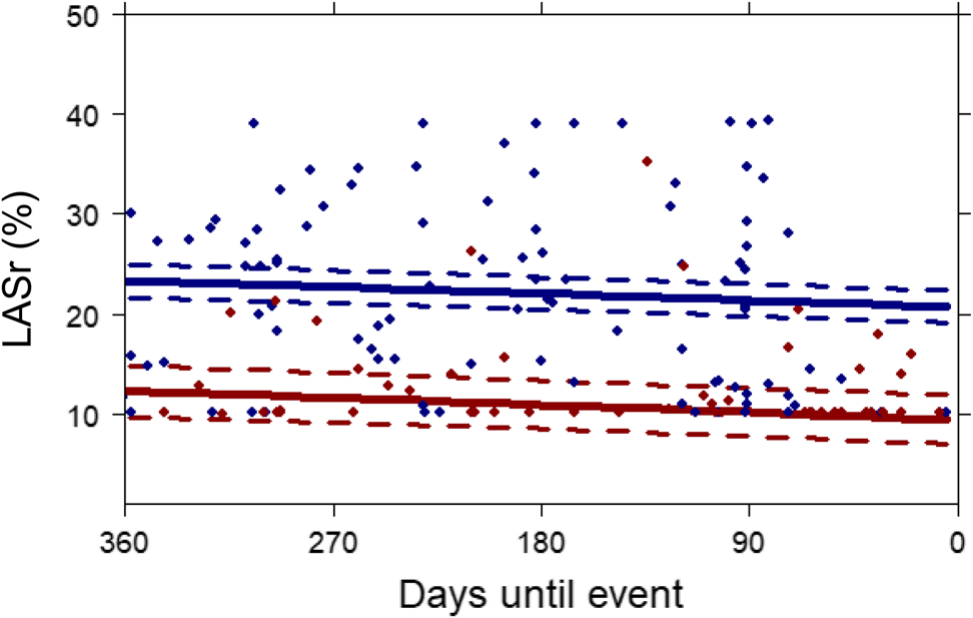
Mean temporal patterns of LA reservoir strain until occurrence of the primary endpoint or censoring. *LASr* left atrial reservoir strain; Continuous lines represent mean temporal patterns for patients with the PEP (red) and patients who remained PEP-free (blue), as extracted from the joint model. Time-point zero represents the occurrence of an event in the PEP patients and censoring in patients who remained PEP-free. Dotted lines represent 95% confidence intervals. Each dot represents a single measurement

In the total population, there was a decrease in LASr over time as the PEP or censoring approached (Beta [95% CI] − 1.72 [− 2.46 to − 0.98]) per LASr (%) change per year). Supplementary Fig. 2 and Fig. 3 show the temporal evolution of patients who experienced the PEP and those who did not. Although, as described above, repeatedly measured LASr was associated with the occurrence of the PEP, and the average LASr was lower in patients who experienced the PEP compared to those who did not, this difference remained stable over time. LASr did not diverge further between patients with vs. without the PEP, as the PEP or censoring approached.

## Discussion

We demonstrated that during a median follow-up of 2.5 years, repeated measurements of LASr were significantly associated with adverse cardiovascular events in HFrEF patients, independent of repeatedly measured GLS, LAVI, *E/A* ratio, *E/e*’ ratio, and NT-proBNP. LASr was a stronger predictor than GLS, LAVI, *E/A* ratio, and *E/e*’ ratio. Although repeated measurements of LASr were associated with the primary outcome, the difference in LASr remained stable over time, and temporal LASr evolutions did not further diverge in patients with events versus those without events. Therefore, for clinical purposes, repeated measurements of LASr do not seem to provide additional value over single measurements over a time frame of 2.5 years. To our knowledge, this is the first study that investigated the prognostic value of repeated LASr measurements in HFrEF.

### Echocardiographic determinants of survival in patients with HFrEF

Although significant improvements in HF therapy have been made in the last two decades, the mortality and morbidity due to HF remain substantial [24]. Numerous multivariable risk models have been proposed to identify patients with a poor prognosis, but the usefulness of these models in clinical practice has been limited [24]. Most of these risk scores incorporate parameters of systolic function. Yet, the assessment of diastolic function may be equally important, as diastolic parameters provide a non-invasive estimation of LAP [4]. However, in a substantial part of HFrEF patients, guideline-based estimation of LAP is not possible, as crucial parameters are often affected by mitral regurgitation (MR) and/or atrial fibrillation (AF) [4]. In addition, some of the conventional parameters that are used for diastolic function in HFrEF have several limitations. For instance, LAVI, which is widely used as an indicator of LAP, does not always provide an accurate estimation of LAP, as LAVI can be increased in the presence of normal filling pressures (e.g. healthy athletes). A study by Benfari et. al. demonstrated that E/e’ ratio outperformed other diastolic parameters as a prognosticator in HFrEF patients, but LA strain was not included in their study[25]. The use of LASr as a non-invasive estimate for LAP has recently gained more interest, as LASr has been shown to be correlated with invasively measured LV filling pressures and LASr showed better prognostic performance than LAVI and *E/e*’ ratio [7, 13–15]. Our results are in line with these previous studies, as we show that LASr is a strong predictor of adverse cardiovascular events; and a stronger predictor than other echocardiographic parameters, such as *E/e*’. In contrast, in our study LAVI was not associated with an increased risk of PEP, which is similar to a study by Modin et al. [26]. This could in part be explained by the fact that LASr is a more sensitive parameter than a volumetric parameter such as LAVI, and that an impairment in LA function is detected earlier than changes in LA volume [27].

Our study confirms and extends previous evidence on the added prognostic value of LASr in HFrEF. A few previous studies have investigated the prognostic value of single measurements of LASr in HFrEF patients [13–15]. In a study consisting of 405 patients with an LVEF < 40%, LASr strongly predicted adverse outcomes, independent of other clinical and echocardiographic predictors of prognosis [13]. A study by Malagoli et al. showed similar results; patients with lower LASr showed worse event-free survival than those with higher LASr [14]. In acute HF, LASr was also shown to be a significant prognosticator [15]. These studies only examined baseline measurements of LASr. Our study is the first to investigate the prognostic value of repeatedly measured LASr, and its added value over a single baseline LASr assessment, and over repeated measurements of other echocardiographic variables. We showed that repeated measurements of LASr were associated with the PEP, and the association persisted after consecutively adding repeated measurements of GLS, LAVI, *E/A* ratio, *E/e*’ ratio, and NT-pro-BNP. However, the difference in LASr between patients with events versus those without events remained stable over time, and temporal LASr evolutions did not further diverge as the PEP or censoring approached. A factor that may in part have contributed to these findings, is that in advanced stages of HF further reduction of already low values of LASr is less likely.

### Results in the context of LA physiology and function in HFrEF

The LA plays a pivotal role in the filling of the LV and contributes to the cardiac output as the LA interacts with the LV and the pulmonary veins. The LA cycle is composed of three phases, which reflect the three main LA functions, reservoir, conduit, and contractile function [20]. A recent meta-analysis found a normal value of > 39% for LASr in healthy individuals [28, 29]. Mean LASr in our population of HFrEF patients was 20.9%. Therefore, profound LA dysfunction exists in our cohort of HFrEF patients, which is in line with previous literature [28].

In the cardiac cycle, LASr and GLS are tightly coupled, as a maximal expansion of the LA takes place during LV systole. This is supported by the observation that LASr and GLS are significantly correlated in HFrEF [7]. Our results confirm this, as a more advanced impairment of GLS was significantly correlated with an impairment in LASr. Previously, we have demonstrated that baseline and repeated measurements of GLS provide incremental prognostic value over LVEF [30]. In the current investigation, we observed that LASr outperforms GLS as a prognostic marker in chronic HFrEF patients. This finding is in line with previous studies that have shown that LASr was superior in predicting outcomes compared to GLS [31]. A potential explanation is that LASr might be affected by atrial inflammation and atrial fibrosis, which restricts atrial stretching, independent of LV longitudinal contraction and subsequent impairment of GLS. Our study is the first to report that repeated measurements of LASr were associated with clinical outcomes, independent of repeated measurements of GLS. Our results extend and add to previous studies and underline that LASr has more value as a prognostic marker in clinical practice than GLS, as well as other known prognostic markers, in stable patients with chronic HFrEF.

### Study limitations

Several limitations of our study should be noted. First, treating physicians were not blinded to the conventional parameters assessed by echocardiography and therefore echocardiographic characteristics might have influenced treatment. However, LASr values were not available for the treating physicians as these were assessed retrospectively. Secondly, the sample size of the study was modest and so was the number of endpoints. To prevent overfitting, we fitted multiple multivariable models containing different confounders. In addition, we adjusted for the duration of HF at baseline, to control for possible lead-time or length–time bias. Lastly, our cohort consisted of patients who were relatively young and in NYHA class I and II. Our results can therefore not be extrapolated to older patients in a more advanced stage of HF.

## Conclusion

Repeatedly measured LASr was significantly associated with adverse cardiovascular events in patients with HFrEF. However, although the temporal trajectories of LASr were different in patients who reached the PEP compared to those who did not, they did not diverge as the PEP or censoring approached, and therefore repeatedly measuring LASr does not seem to provide additional incremental prognostic information over a single baseline measurement over a median follow-up time of 2.5 years. A single measurement of LASr showed a stronger prognostic value than conventional echocardiographic parameters. Therefore, LASr should be considered for routine use in clinical practice in patients with HFrEF, for prognostication and potentially for guiding treatment.

## Supplementary Information

Below is the link to the electronic supplementary material.

Supplementary file1 (DOCX 373 KB)
