# Supplementary material for: Prognostic value of temporal patterns of left atrial reservoir strain in patients with heart failure with reduced ejection fraction
Source: Clin Res Cardiol. 2023 Jun 13;113(9):1306–16. doi: 10.1007/s00392-023-02244-x (PMC11371887; doi:10.1007/s00392-023-02244-x)
Supplement: Supplementary file 1 — Supplementary file1 (DOCX 373 KB) [file 392_2023_2244_MOESM1_ESM.docx]

**Prognostic value of temporal patterns of left atrial reservoir strain in patients with heart failure with reduced ejection fraction**

***Supplementary material***

S. Abou Kamar^1,2,3^*, Y.S. Aga^1,3^*, M. de Bakker^1^, V.J van den Berg^1,4,5^, M. Strachinaru^1^, D. Bowen^1^, R. Frowijn^1^, K.M. Akkerhuis^1^, J.J. Brugts^1^, O. Manintveld^1^, V. Umans^4^, M. Geleijnse^1^, R.A. de Boer^1^, E. Boersma^1^, I. Kardys^1^**, B.M. van Dalen^1,3^**

*Shared first author

**Shared last author

1. Department of Cardiology, Thoraxcenter, Erasmus University Medical Center, Rotterdam, The Netherlands
2. The Netherlands Heart Institute, Utrecht, The Netherlands
3. Department of Cardiology, Franciscus Gasthuis & Vlietland, Rotterdam, The Netherlands
4. Department of Cardiology, Northwest Clinics, Alkmaar, The Netherlands
5. Department of Anesthesiology, Leiden University Medical Center, Leiden, The Netherlands

**Address for correspondence and proofs:**

Prof. Dr. Isabella Kardys

Department of Cardiology, Erasmus MC, University Medical Center Rotterdam,

Room Na‐316, P.O. Box 2040, 3000 CA Rotterdam, the Netherlands.

E‐mail: [i.kardys@erasmusmc.nl](mailto:i.kardys@erasmusmc.nl)

**Table 1** Associations of baseline and repeatedly measured LASr with the primary endpoint.

|  | SR (*N=105*) | | AF (*N=41*) | | MR (*N=37*) | | No MR(*N=121*) |  |
| --- | --- | --- | --- | --- | --- | --- | --- | --- |
|  | HR(95% CI) | P value | HR(95% CI) | P value | HR(95% CI) | P value | HR(95% CI) | P value |
| Baseline measurements* | | | | | | | |  |
| LASr | 0.35(0.16-0.77) | 0.008 | 0.64(0.23-1.79) | 0.4 | 0.74(0.26-2.16) | 0.6 | 0.42(0.21-0.83) | 0.1 |
| GLS | 0.44(0.21-0.91) | 0.03 | 0.93(0.45-1.94) | 0.9 | 0.83(0.37-1.87) | 0.7 | 0.57(0.33-1.00) | 0.05 |
| LAVI | 0.67(0.41-1.08) | 0.1 | 0.93(0.63-1.38) | 0.7 | 1.04(0.65-1.66) | 0.9 | 0.77(0.54-1.11) | 0.2 |
| E/A ratio | 0.69(0.46-1.03) | 0.07 | 0.55(0.23-1.28) | 0.2 | 0.77(0.42-1.42) | 0.4 | 0.67(0.41-1.09) | 0.1 |
| E/e’ ratio | 0.71(0.45-1.12) | 0.1 | 0.74(0.47-1.17) | 0.2 | 0.36(0.16-0.84) | 0.02 | 0.79(0.56-1.11) | 0.2 |
| Repeated measurements of LASr | | | | | | | |  |
| Model 1 | 0.13(0.04-0.33) | <0.001 | 0.05(0.01-0.39) | <0.001 | 0.08(0.02-0.25) | <0.001 | 0.17(0.10-0.39) | <0.001 |
| Model 2 | 0.15(0.06-0.34) | <0.001 | 0.03(0.01-0.27) | <0.001 | 0.09(0.03-0.22) | <0.001 | 0.14(0.06-0.33) | <0.001 |

*Corrected for age, sex, duration of HF, baseline NT-proBNP

Model 1: corrected for age, sex, duration of HF

Model 2: corrected for age, sex, duration of HF, NT-proBNP

Further multivariable adjustment was not possible because of limited number of primary endpoints in the subgroups with AF and MR.

SR, sinus rhythm; AF, atrial fibrillation; MR, mitral regurgitation; LASr, left atrial reservoir strain; GLS, global longitudinal strain; LAVI, left atrial volume indexed; E/A ratio, the ratio of the peak early left ventricular filling velocity over the late filling velocity; E/e’ ratio, E to early diastolic mitral annular tissue velocity;

**Supplementary figure 1** Scatterplots for LASr and variables of interest.


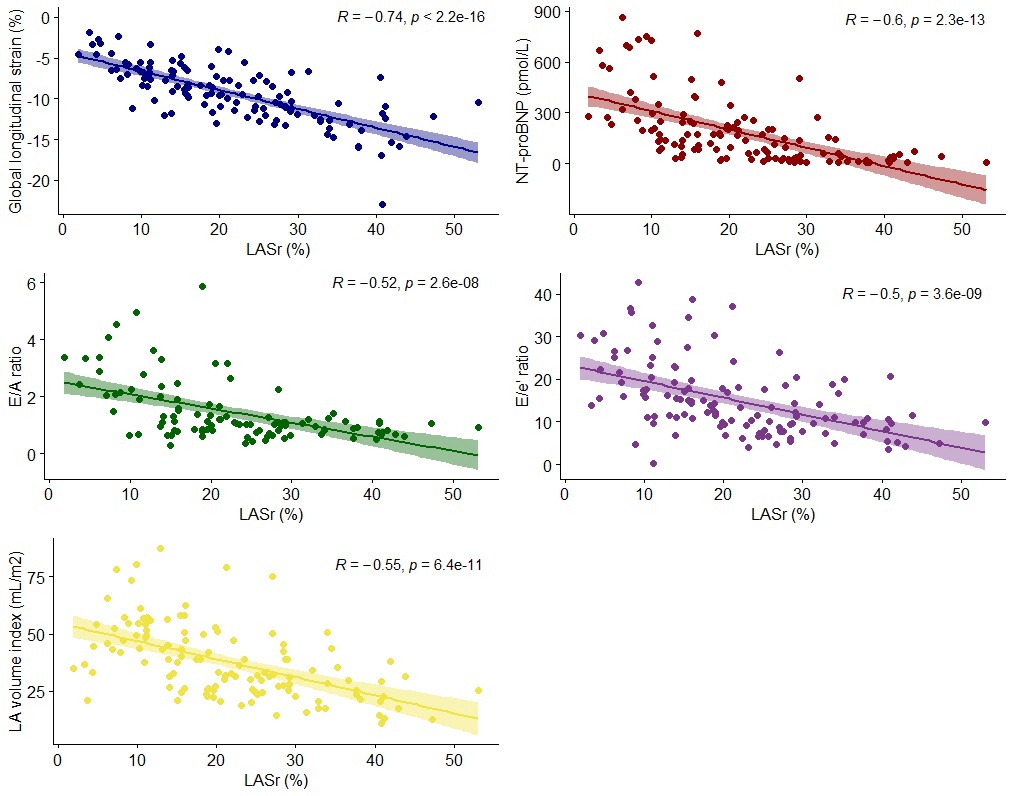


LASr, left atrial reservoir strain; E/A ratio, the ratio of the peak early left ventricular filling velocity over the late filling velocity; E/e’ ratio, E to early diastolic mitral annular tissue velocity;. R= correlation coefficient. Regression lines are provided for the variables of interest.

**Supplementary figure 2** Medians of first and last available values of variables of interest, according to endpoint status.


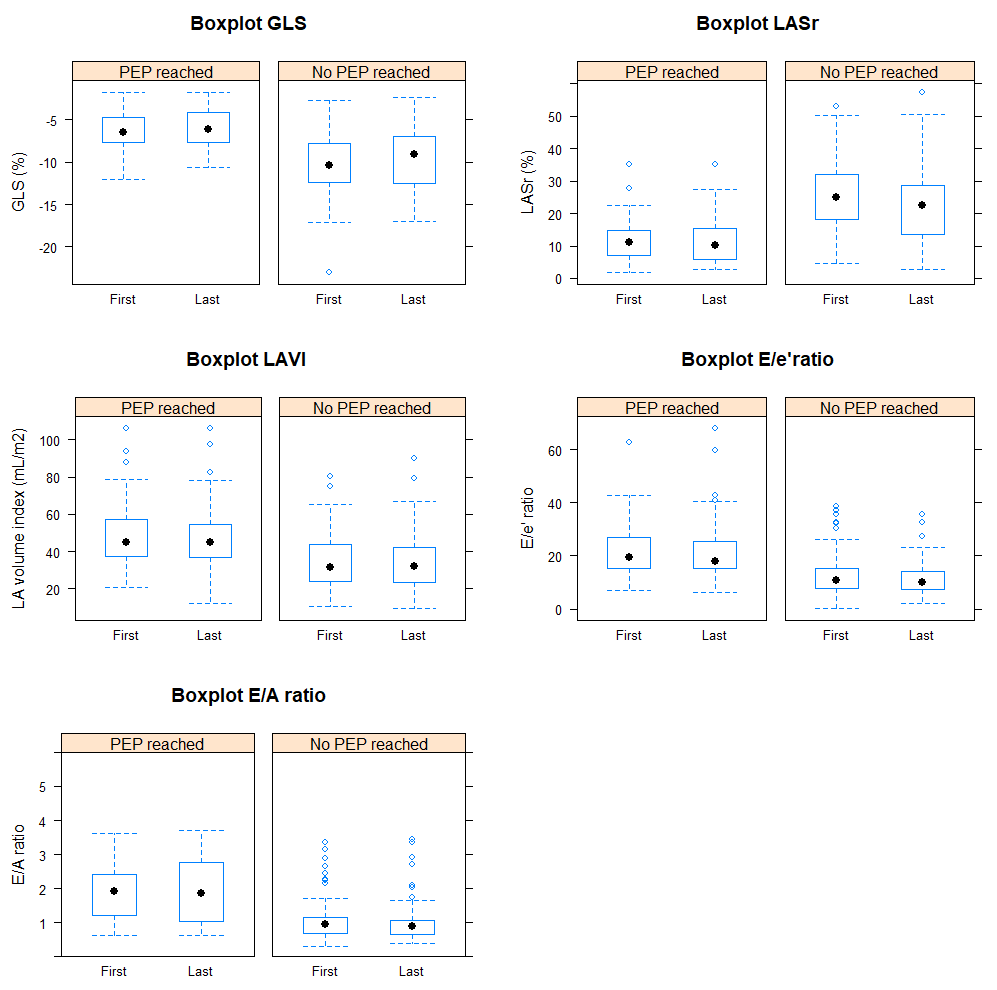


PEP, primary endpoint; LASr, left atrial reservoir strain; GLS, global longitudinal strain; E/A ratio, the ratio of the peak early left ventricular filling velocity over the late filling velocity; E/e’ ratio, E to early diastolic mitral annular tissue velocity; LAVI, left atrial volume index. The boxplots show the average values at the first and last available measurements.
